# Adenosine-to-inosine RNA editing in cancer: molecular mechanisms and downstream targets

**DOI:** 10.1093/procel/pwae039

**Published:** 2024-08-10

**Authors:** Hao Cheng, Jun Yu, Chi Chun Wong

**Affiliations:** Institute of Digestive Disease and Department of Medicine and Therapeutics, State Key Laboratory of Digestive Disease, Li Ka Shing Institute of Health Sciences, The Chinese University of Hong Kong, Hong Kong, SAR 518172, China; Institute of Digestive Disease and Department of Medicine and Therapeutics, State Key Laboratory of Digestive Disease, Li Ka Shing Institute of Health Sciences, The Chinese University of Hong Kong, Hong Kong, SAR 518172, China; Institute of Digestive Disease and Department of Medicine and Therapeutics, State Key Laboratory of Digestive Disease, Li Ka Shing Institute of Health Sciences, The Chinese University of Hong Kong, Hong Kong, SAR 518172, China

**Keywords:** adenosine-to-inosine (A-to-I), RNA modification, ADARs, ADATs, cancer

## Abstract

Adenosine-to-inosine (A-to-I), one of the most prevalent RNA modifications, has recently garnered significant attention. The A-to-I modification actively contributes to biological and pathological processes by affecting the structure and function of various RNA molecules, including double-stranded RNA, transfer RNA, microRNA, and viral RNA. Increasing evidence suggests that A-to-I plays a crucial role in the development of human disease, particularly in cancer, and aberrant A-to-I levels are closely associated with tumorigenesis and progression through regulation of the expression of multiple oncogenes and tumor suppressor genes. Currently, the underlying molecular mechanisms of A-to-I modification in cancer are not comprehensively understood. Here, we review the latest advances regarding the A-to-I editing pathways implicated in cancer, describing their biological functions and their connections to the disease.

## Introduction

RNA, like DNA and proteins, can be modified by a variety of enzymes. RNA modifications impact RNA processing, stability, and translation, which have led to the recent development of a new field of research known as epitranscriptomics (the study of RNA modifications) (Roundtree and He, 2016; Saletore et al., 2012). To date, there are over 170 different types of RNA modifications that have been identified, and the development of high-throughput sequencing technologies has enabled the mapping of these modification in healthy and disease states (Boccaletto et al., 2018; Roundtree et al., 2017). All known RNA species, including messenger RNA (mRNA), ribosomal RNA (rRNA), transfer RNA (tRNA), microRNA (miRNA), and long noncoding RNA (lncRNA), have been shown to be modified. All four RNA bases and the ribose sugar could be targets of modification (Barbieri and Kouzarides, 2020; Li and Mason, 2014; Roundtree et al., 2017). Adenosine-to-inosine (A-to-I) RNA editing, in which adenosine (A) is converted to inosine (I), is one of the most prevalent RNA modifications. It was first detected over three decades ago in frog eggs and embryos as an activity that unwound RNA duplexes before researchers realized that adenosine residues in RNA have been converted to inosines (Rebagliati and Melton, 1987; Tan, 2023; Zinshteyn and Nishikura, 2009). This is followed by discovery of adenosine deaminase acting on RNA (ADAR) catalyzing A-to-I modifications (Wagner et al., 1989). Initially, a limited number of editing sites were discovered serendipitously in the protein-coding regions of mRNAs through comparing human genomic DNA versus cDNA sequences. With advances in next-generation and bioinformatic tools, it is possible to decipher A-to-I RNA editing globally, leading to a number of significant breakthroughs in the last decade. Surprisingly, the most frequent and widespread targets of A-to-I RNA editing are double-stranded RNAs (dsRNAs) made from inverted Alu repetitive elements (IR-Alu dsRNAs), which are located within introns and untranslated regions (Bazak et al., 2014; Peng et al., 2012; Porath et al., 2014). Moreover, inosine is frequently detected in tRNAs or viral RNAs, but seldom in DNA (Grosjean et al., 1996; Polson et al., 1996). Recent studies have demonstrated that A-to-I is mainly catalyzed by three groups of RNA adenosine deaminases. Apart from aberrant regulation of A-to-I regulators (ADARs) (Bass, 2002; Eisenberg and Levanon, 2018; Nishikura, 2010), adenosine deaminase tRNA-specific family (ADATs) catalyzes A-to-I conversion on tRNAs, whereas the testis-specific adenosine deaminase domain-containing (ADAD) protein family is essential for male fertility (Dai et al., 2023; Islam et al., 2023; Lu et al., 2023).

In this review, we will highlight the most recent developments in the field of A-to-I RNA modification, with a special emphasis on the roles of A-to-I in the genesis and progression of cancer, as well as the potential future directions of A-to-I research.

## Regulators of A-to-I RNA modification

### ADAR1/ADAR2/ADAR3

The family of ADARs was identified unintentionally by Bass and Weintraub (1988) as the enzymes responsible for “denaturing” dsRNA in *Xenopus laevis* embryos and unwittingly thwarting RNA interference (RNAi). Since then, ADARs (formerly referred to as DRADAs or DSRADs) have emerged as regulators of the gene expression output of a cell, and they are often deregulated in cancers.

In mammals, there are three ADAR genes, including ADAR1 (also known as DRADA), ADAR2 (also known as ADARB1), and ADAR3 (also known as ADARB2), with distinct differences in structure, location, and function (Shen et al., 2022; Xu and Öhman, 2018). ADAR1 has been demonstrated to be expressed and catalytically active in most tissues and encodes two isoforms generated from alternate promoter usage, a short, constitutive, and nuclear-restricted ADAR1 p110 isoform, and a longer, interferon (IFN)-inducible ADAR1 p150 isoform that is present in both the nucleus and cytoplasm (Hogg et al., 2011; Patterson and Samuel, 1995). ADAR2 has a more targeted expression pattern to tissues such as the brain, lungs, and arteries, and is responsible for the notably higher A-to-I editing rates in neuronal tissues (Baker and Slack, 2022). ADAR3, on the other hand, is specifically expressed in the central nervous system and has yet to show detectable editing action, although it may inhibit the activity of other ADARs in the brain (Chen et al., 2000; Oakes et al., 2017; Raghava Kurup et al., 2022; Tan et al., 2017; Walkley and Li, 2017; Zhang et al., 2018).

The functional domains of ADARs are shared. Each ADAR harbors two or three dsRNA-binding domains (dsRBDs) (~65 amino acids) with an α‑β‑β‑β‑α configuration that interacts directly with dsRNA (Stefl et al., 2006). The deaminase domain, which is crucial for its A-to-I editing activity, lies in the carboxy-terminal region. In addition, all ADARs contain a nuclear localization signal (NLS), which is responsible for the trafficking of ADAR1 to the nucleus and nucleolus; however, ADAR1 p150 isoform includes a nuclear export signal (NES) that allows it to shuttle between nucleus and cytoplasm (Desterro et al., 2003; Nishikura, 2016; Strehblow et al., 2002). Some structural characteristics are specific to individual ADAR members. For instance, ADAR1 has Z-DNA-binding domains (Zα and Zβ) (Herbert et al., 1997), whereas ADAR3 contains an Arg-rich single-stranded RNA (ssRNA)-binding R domain at its amino terminus (Chen et al., 2000). The deaminase domains of ADAR1 and ADAR2 can catalyze the hydrolytic deamination of adenosine-to-inosine in dsRNA. Inosine preferentially couples with cytidine (C) over uridine (U), and inosine is read as guanosine (G) by the ribosomes, thus enabling the same gene to gain the capacity to express various protein isoforms (Licht et al., 2019; Zinshteyn and Nishikura, 2009). Thus, ADARs increase protein diversity at posttranscriptional level, fine-tune regulation of gene expression, and enhance potential adaptability (Gommans et al., 2009).

**Figure 1.**
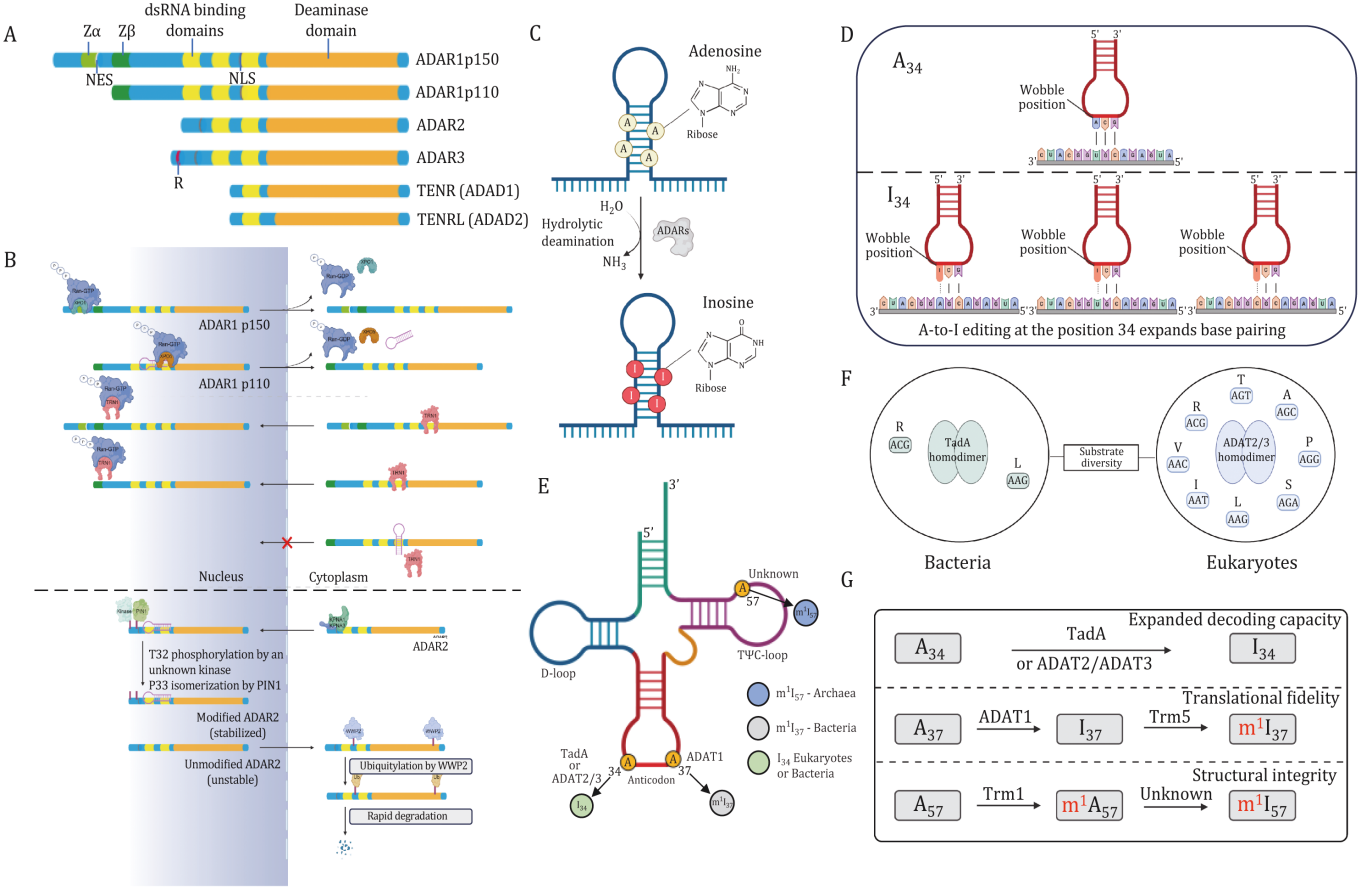
Deamination of adenosine-to-inosine by ADAR, tRNA (ADAT) and ADAD proteins and cellular localization of ADAR1 and ADAR2. (A) Members of the human ADAR family (ADAR1, ADAR2, and ADAR3) and ADAD family (TENR and TENRL) share common functional domains. These include two or three repeats of the dsRBD and a catalytic deaminase domain. Certain ADAR members possess unique structural features, such as Z-DNA-binding domains (Zα and Zβ) and Arg-rich ssRNA-binding R domain. (B) XPO1 binds to NES located within Zα structural domain and regulates nuclear export of ADAR1p150 in the presence of Ran-GTP. Nuclear import of ADAR1p110 is mediated by binding of TRN1 to the third dsRBD. NLS located in the third dsRBD binds with TRN1 to assist in the targeting of ADAR1 in the nucleus (top). Localization of ADAR2 in the nucleus and nucleolus is regulated by the interaction between karyopherin subunit α1 (KPNA1) and KPNA3, which bind to the NLS located in N-terminal region. Phosphorylation of Thr32 enables interaction of ADAR2 with prolyl-isomerase PIN1 in a dsRNA-binding-dependent manner, which isomerizes Pro33 and positively controls the nuclear localization and stability of ADAR2. E3 ubiquitin ligase WWP2 promotes rapid degradation of ADAR2 in cytoplasm (bottom). (C) Adenosine deaminases acting on RNA convert adenosine to inosine via hydrolytic deamination. (D) The formation of inosine 34 in tRNA. Anticodon pairing for unmodified (left, U-ended codons) and modified (right, U-, C-, and A-ended codons) tRNAs. (E) Schematic diagram of the cloverleaf tRNA secondary structure. (F) Substrate diversity of tRNA-specific deaminases in bacteria (TadA) and eukaryotes (ADAT2/3). (G) Diverse functions of A-to-I modifications in tRNAs. D-loop, dihydrouridine-loop; SAH, S-adenosyl-L-homocysteine; SAM, S-adenosyl-L-methionine; TadA, tRNA adenosine deaminase A; TrmI, tRNA methyltransferase; Trm5, tRNA methyltransferase 5; TΨC-loop, ribothymine-pseudouracyl-cytosine-loop. Ub, ubiquitin.

Localization of ADARs is tightly regulated. Although ADAR1 p150 is generally localized in the cytoplasm and ADAR1 p110 is predominantly located in the nucleus, both of them can shuttle between the nucleus and cytoplasm (Desterro et al., 2003; Jarmoskaite and Li, 2024; Strehblow et al., 2002). Nuclear export of ADAR1 p150 is mediated by binding of the nuclear export factor exportin 1 (XPO1; also known as CRM1) to NES located within the Zα domain, together with Ran-GTP (Karki et al., 2021; Nishikura, 2016; Poulsen et al., 2001). Meanwhile, binding of transport protein 1 (TRN1) to the third dsRBD (containing NLS) mediates nuclear import of ADAR1 p110, a process inhibited by dsRNA binding; whereas nuclear export of ADAR1 p110 is regulated by XPO5–Ran-GTP and is mediated by dsRNA binding to dsRBDs (Fritz et al., 2009; Sakurai et al., 2017; Strehblow et al., 2002). As a consequence, dsRNA binding promotes a net efflux ADAR1 p110 from nucleus (Barraud et al., 2014). The predominantly nucleolar localization of ADAR2 is regulated by the binding of karyopherin subunit α1 (KPNA1) and KPNA3 to an Arg‑rich NLS in N‑terminal region (Ashley et al., 2024; Behm et al., 2017; Maas and Gommans, 2009). The nuclear localization and stability of ADAR2 are regulated by posttranslational modifications. Phosphorylation of Thr32 activates ADAR2 interaction with the prolyl‑isomerase PIN1 in a dsRNA‑binding-dependent manner, which isomerizes Pro33 and positively controls the nuclear localization and stability of ADAR2 (Eisenberg and Levanon, 2018; Keegan et al., 2023). In contrast, it has been observed that the E3 ubiquitin ligase WWP2 has a role in promoting the degradation of ADAR2 in the cytoplasm, which is why ADAR2 is usually not detected in the cytoplasm (Fig. 1A–C) (Marcucci et al., 2011; Vesely and Jantsch, 2021; Walkley and Li, 2017).

### ADAT1/ADAT2/ADAT3

The A-to-I RNA editing processes have also been observed in tRNA. Methylinosine 37 (m^1^I_37_) is exclusive to eukaryotic tRNA^Ala^ and is generated by a two-step process, first involving the A-to-I modification catalyzed by homologous ADAT1 dimer, which belongs in the evolutionary clade containing ADARs at Position 37 in the anticodon loop, followed by methylation by tRNA methyltransferase 5 (Trm5) (Björk et al., 2001; Jühling et al., 2009; Maas et al., 1999). Methylinosine 37 modification is thought to repress translational frameshifts and enhance translational precision (Grosjean et al., 1996; McKenney et al., 2017).

Modification of A_34_ of tRNAs is abundant in both bacteria and eukaryotes but nonexistent in archaea (Dixit et al., 2019). In bacteria, A_34_-to-I_34_ deamination reaction is present in two different tRNAs (mostly on tRNA^Arg^_ACG_ and seldom on tRNA^Leu^_AAG_) and modification is catalyzed by the homodimeric tRNA adenosine deaminase A (TadA), the ancestor enzyme of all tRNA-specific deaminases (Rafels-Ybern et al., 2019; Wolf et al., 2002). In contrast, eukaryotic I_34_ is found in eight tRNAs (tRNA^Leu^_AAG_, tRNA^Pro^_AGG_, tRNA^Ala^_AGC_, tRNA^Val^_AAC_, tRNA^Ser^_AGA_, tRNA^Ile^_AAT_, tRNA^Thr^_AGT_, and tRNA^Arg^_ACG_), and the A-to-I conversion is catalyzed by the heterodimeric adenosine deaminase consisting of catalytic subunit ADAT2 and tRNA-binding subunit ADAT3 (hetADAT). A conserved proton-shunting glutamate that is required for catalytic activity of ADAT2 has been lost in ADAT3, leaving this subunit inactive (Elias and Huang, 2005; McKenney et al., 2017).

Interestingly, inosine is the terminal modified base at Position 34, while at Positions 37 and 57 inosine could be further modified to a methylated state (m^1^I_37_, m^1^I_57_, or m^1^Im_57_) (Jühling et al., 2009; Machnicka et al., 2013). I_34_-tRNAs are prone to internal cleavage by human endonuclease V, a highly conserved ribonuclease that specifically cleaves inosine-modified tRNAs at the anticodon (Vik et al., 2013). Stress conditions, such as oxidation and starvation, can trigger the cleavage of tRNAs within the anticodon loop as a part of the cellular response and the resultant fragments perform a variety of regulatory functions that are still largely unknown (Lyons et al., 2018; Phizicky and Hopper, 2010).

Adenosines at Position 57 (A_57_) is converted into m^1^I_57_ in a two-step reaction. In the first phase, A_57_ at TΨC-loop is methylated by an S-adenosyl-L-methionine (SAM)-dependent tRNA methyltransferase (TrmI) (Droogmans et al., 2003). Then, m^1^A_57_ is catalyzed to m^1^I_57_ by an enzyme that has not yet been identified (Grosjean et al., 1995). A_57_ is also reported to be altered into di-methylated inosine (1,20-O-dimethylinosine, m^1^Im_57_) in hyperthermophilic species (Edmonds et al., 1991). Methylinosine 57 (m^1^I_57_ or m^1^Im_57_) is only observed in archaeal tRNA^Ile^ and its function is yet unknown (Fig. 1D–G).

### ADAD1/ADAD2

The testis-specific ADAD protein family, including ADAD1 and ADAD2, are essential for male fertility. Specifically, ADAD1 is expressed in haploid spermatids, whereas ADAD2 is expressed in mid- to late-pachytene spermatocytes. *Adad1*^−/−^ male mice showed reduced sperm counts, decreased motility and malformed heads; deletion of *Adad2* in mice resulted in male sterility, as *Adad2*^−/−^ germ cells are unable to progress beyond round spermatids (Connolly et al., 2005; Dai et al., 2023; Snyder et al., 2020). However, analysis of ADAD adenosine deaminase domain suggests that they are likely to be catalytically inactive. Thus, they might function as negative regulators of RNA editing in male germ cells.

## Function of A-to-I editing in RNAs

ADAR-mediated editing has been identified at diverse RNA species, which could confer differentially effects on their function. A-to-I editing in the coding region can produce non-synonymous mutations, while A-to-I editing in the introns or 3ʹ-UTRs could modulate the expression levels of related coding regions. The majority of A-to-I editing sites are found in introns and 3ʹ-UTRs of coding genes, with 1% or less occurring in coding exons (Bahn et al., 2012; Picardi et al., 2015; Wang et al., 2013a). In ncRNAs, editing affects ncRNAs maturation and targeting, whilst A-to-I editing of tRNAs at wobble base at Position 34 is essential for decoding the redundancy of the genetic code.

### Function of A-to-I editing in mRNA

**Figure 2.**
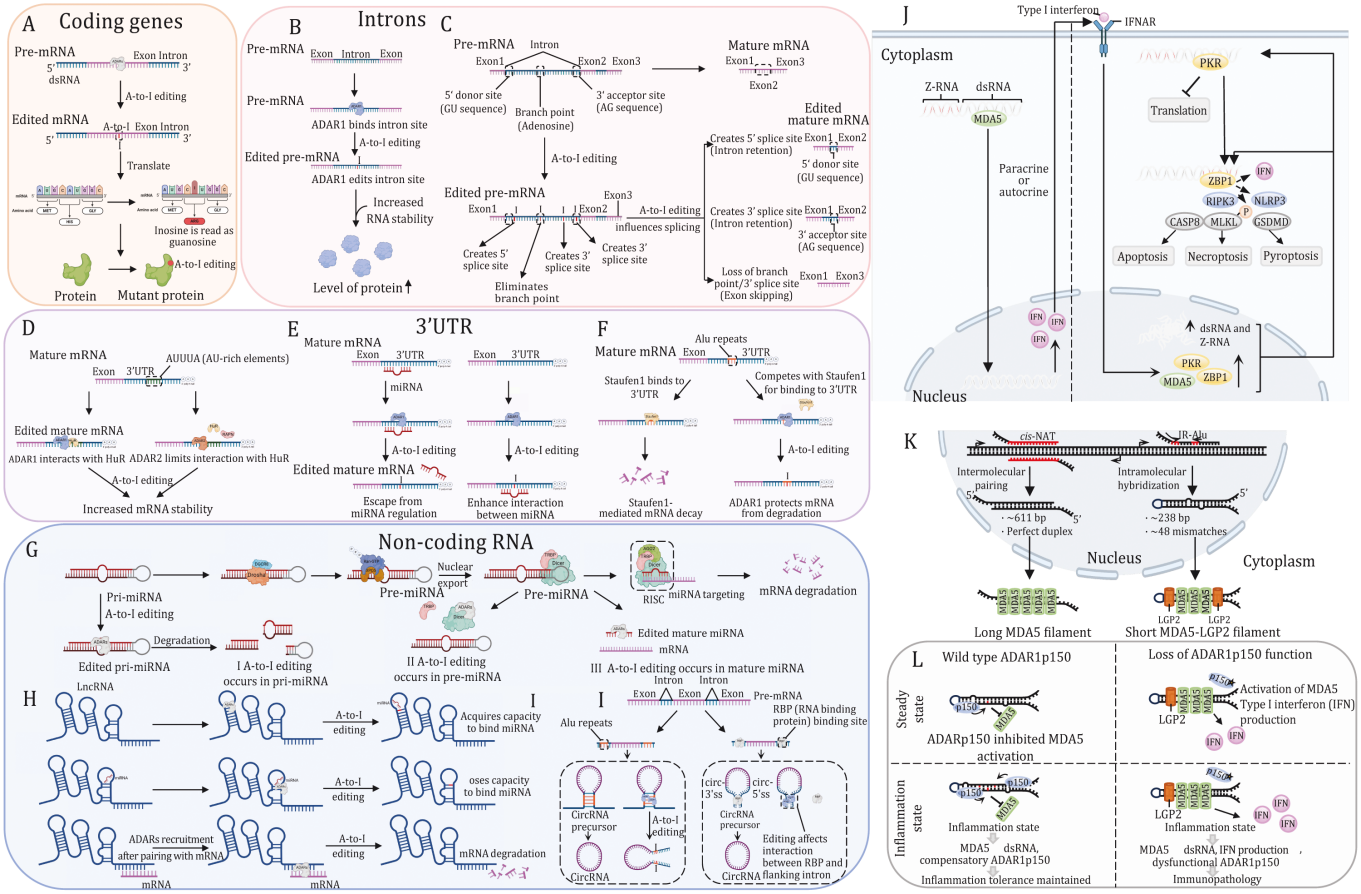
Regulatory mechanisms for RNA editing-dependent and -independent roles of ADARs and the function of ADAR1p150 isoform in regulating innate immune and cell death responses to dsRNA. (A) In coding regions, A-to-I editing leads to amino acid changes (recoding). (B) A-to-I editing in the intron of pre-mRNA results in stabilization of mRNA and abundance of protein. (C) A-to-I editing of donor sites, acceptor sites, and branch points in pre-mRNA intron plays a role in pre-mRNA alternative splicing. (D) ADAR1 and ADAR2 interact with human antigen R (HuR) proteins to co-regulate common transcripts. (E) By creating or disturbing the binding site of the miRNAs, editing of the 3ʹUTR can alter mRNA stability. Loss of the ability to bind miRNA (left); acquired the ability to bind miRNA (right). (F) ADAR1 competes with Staufen1 protein for dsRNA-binding sites and antagonize Staufen1-induced mRNA decay of antiapoptotic genes. (G) Primary miRNA (pri-miRNA) transcripts fold to form dsRNA structures, which are processed in the nucleus into precursor-miRNAs (pre-miRNAs) of ~70 nucleotides in length by the RNase III protein Drosha, in complex with the pri-miRNA recognition factor DGCR8. Pre-mRNAs are then exported to the cytoplasm, where they are processed further by another RNase III protein, Dicer, in complex with TRBP to generate double-stranded, mature miRNAs of ~22 nucleotides in length. Mature miRNAs are loaded onto Argonauteproteins and together form the core of the RISC. The miRNA guide strand directs the RISC to the target mRNAs, causing translation repression or mRNA decay. The A-to-I editing may affect the process at three different points. I: in pri-miRNA, editing occurs at the RNA site that binds to Drosha and DGCR8, which causes the formation of fragmented pri-miRNA; II: in pre-miRNA, ADAR1 interacts with Dicer to abort the miRNA biosynthesis process; III: in mature miRNA, editing occurs at the RNA site that binds to mRNA. (H) Regulatory mechanisms of A-to-I editing for dsRNA on lncRNA. Acquired the ability to bind miRNA (upper); loss of the ability to bind miRNA (middle); lncRNA-mRNA interaction may facilitate target mRNA decay. (I) A-to-I editing of Alu region can hinder circRNA biogenesis (left) and ADAR1 can influence the binding of RBPs to adjacent intron regions, leading to change in circRNA formation (right panel). (J) Upon ADAR1 depletion, unedited dsRNA triggers the pattern recognition receptor MDA5, PKR and unedited Z-RNA triggers ZBP1, both ultimately leading to interferon-induced and apoptotic, necroptotic and pyroptotic antiviral mechanisms. (K) Intermolecular pairing of cis-natural antisense transcripts (cis-NATs) or intramolecular hybridization of inverted repeat Alu (IR-Alu) elements generates endogenous MDA5 agonists. Multiple MDA5 proteins, aided by the cofactor laboratory of genetics and physiology 2 (LGP2), cooperatively bind to double-stranded cis-NATs or IR-Alu sequences, generating filaments containing active MDA5. (L) At steady state, ADAR1p150 prevents MDA5 filament formation by A-to-I editing and sequestration of endogenous dsRNA (upper left panel). Type I interferon signaling during inflammation increases MDA5 expression and increases the concentration of endogenous dsRNA. Simultaneous upregulation of ADAR1p150 isoform leads to an increase in A-to-I editing and sequestration of self-dsRNA to maintain tolerance (upper right panel). Loss of ADAR1p150 function results in the accumulation of immunostimulatory endogenous dsRNA that triggers MDA5 activation and Type I interferon production (lower left panel). This, in turn, results in activation of a MDA5-Type I interferon-mediated positive-feedback loop. Increased expression of mutant ADAR1p150 is unable to inhibit this process, resulting in immunopathology (lower right panel).

A-to-I editing of mRNA can occur in exons, intron, and 3ʹ-UTR regions. There are millions of RNA editing sites in the human transcriptome. Only a very small fraction of those sites is in protein-coding mRNA sequences, with the vast majority in noncoding RNA sequences within untranslated regions and introns (Eisenberg and Levanon, 2018; Ramaswami and Li, 2014). Noncoding RNA editing sites in humans are most frequently observed in IR-Alu repetitive elements in which two adjacent Alu elements in opposite orientations in the same transcript form dsRNAs (Chen et al., 2008; Levanon et al., 2004; Ramaswami et al., 2012). Early mechanistic studies on ADARs mostly focused on protein recoding, which potentially alter amino acid sequence, thereby leading to inactivation or activation of the translated protein (Fig. 2A) (Datta et al., 2023). Where the coding sequence exhibits a double-stranded structure on the exon(s), these regions might be potential targets for ADAR1-mediated A-to-I editing. Several genes have been identified to undergo A-to-I editing in exon(s), such as antizyme inhibitor 1 (AZIN1), bladder cancer-associated protein (BLCAP), gamma-aminobutyric acid A receptor alpha3 (Gabra3), glioma-associated oncogene 1 (GLI1), and integrin a2 (ITGA2). Some intronic regions can also recruit and attach to ADAR1, leading to increased transcript stability and abundance in an A-to-I editing-dependent manner (Fig. 2B) (Amin et al., 2017; Liu et al., 2022). Moreover, A-to-I editing of intronic regions may result in various alternative splicing patterns, including creating donor sites (5ʹ GU splice sites), creating or destroying acceptor sites (3ʹ AG splice sites), or creating altogether new splice sites (Fig. 2C) (Lev-Maor et al., 2007; Mallela and Nishikura, 2012; Sakurai et al., 2010; Wang et al., 2020). A-to-I modifications in 3ʹ-UTR have two major effects on the regulation of gene expression. The first mechanism relies on regulating the stability of the mature mRNA. For example, ADAR1 recruits and interacts with human antigen R (HuR, gene name *ELAVL1*), a family of RNA-binding proteins (RBPs) selectively binds to single-stranded AU-rich RNA sequences to increase transcript stability (Fan and Steitz, 1998; Wang et al., 2013a). ADAR2 enhances mRNA stability by restricting the interaction with RNA-degrading proteins, such as HuR and PARN (poly(A)-specific ribonuclease) (Fig. 2D) (Anantharaman et al., 2017; López de Silanes et al., 2003; Meisner et al., 2004; Song et al., 2016). Another common one is editing of Alu dsRNA on 3ʹ-UTR, which otherwise binds to and is regulated by miRNA in the unedited state. Several studies have reported that RNA editing that occurs in the 3ʹ-UTR could create or destroy miRNA binding sites, thereby altering the mRNA stability of cancer-related genes (Fig. 2E and 2F) (Borchert et al., 2009; Liang and Landweber, 2007; Roberts et al., 2018; Soundararajan et al., 2015; Tomaselli et al., 2013; Yang et al., 2017; Zhang et al., 2016).

### Function of A-to-I editing in noncoding RNA

A-to-I editing affects any of the miRNA biogenesis steps, including processing of primary miRNA (pri-miRNA) into precursor miRNA (pre-miRNA) by DORSHA, the conversion of pre-miRNA into mature miRNA by DICER, and loading of miRNA into RNA-induced silencing complex (RISC). Moreover, A-to-I editing can also impact miRNA target selection. In this way, the editing of certain miRNA precursors results in the altered expression or function of corresponding mature miRNAs (Fig. 2G) (Deiuliis, 2016; Slezak-Prochazka et al., 2010; Tomaselli et al., 2013). Additionally, lncRNA are targets of A-to-I modification. Editing primarily occurs in dsRNA regions on lncRNA by ADAR changes its structure, which affects binding of downstream target miRNAs. Long noncoding RNAs can also recruit ADARs, which edit mRNA and, consequently, might promote target mRNA degradation (Fig. 2H) (Flippot et al., 2019; Nishikura, 2016; Silvestris et al., 2020). Similarly, ADAR1 interacts with inverse complementary dsRNA regions (e.g., Alu repeats) of circular RNAs (circRNAs) to make its production less favorable (Chen and Yang, 2015; Ivanov et al., 2015). A-to-I editing can affect the binding of RBPs to flanking intron regions, resulting in changes in the production of circRNAs (Fig. 2I) (Shevchenko and Morris, 2018; Wang et al., 2023b).

### Function of A-to-I modification in tRNA

tRNA translates the genetic code during protein synthesis and is crucial to the efficiency and accuracy of translation (Klinge and Woolford, 2019). tRNAs fold into a cloverleaf secondary structure and adopt an L-shaped architecture, where nucleobases at Positions 34, 35, and 36 form the anticodon that recognizes complementary codon triplets in mRNA (Kim et al., 1973). Eukaryotic ADAT deaminates A_34_ in multiple tRNAs (eight tRNAs in humans). ADAT activity is important to eukaryotes, given the fact that most of the eukaryotic genomes lack genes that code for G_34_-tRNAs. Functionally, the I_34_-tRNA modification potentiates wobble-pairing flexibility of the anticodon, as I_34_-tRNAs could recognize either A-, C-, and U-ended synonymous codons, whereas A_34_-tRNA could only efficiently combine with U-ended codons (Crick, 1966). The major impact of A_34_-tRNA A-to-I modification is on the efficiencies of protein translation, as codon composition bias and clustering of rare codons (codons with few copy numbers of cognate tRNAs) in regions of mRNAs limit the rate of translation (Kubo and Imanaka, 1989; Parmley and Huynen, 2009). Hence, the translation of genes rich in ADAT-sensitive codons [codons translated by I_34_-tRNAs, amino acids threonine, alanine, proline, serine, leucine, isoleucine, valine, and arginine (TAPSLIVR)] can benefit from the increased decoding capacity of inosine-modified tRNAs (Chan et al., 2015; Lyu et al., 2020; Novoa and Ribas de Pouplana, 2012). In agreement with this prediction, self-renewing embryonic stem cells that express many genes enriched in ADAT-sensitive codons and also displayed enhanced ADAT2 expression (Bornelöv et al., 2019).

## Role of A-to-I in cancer

**Figure 3.**
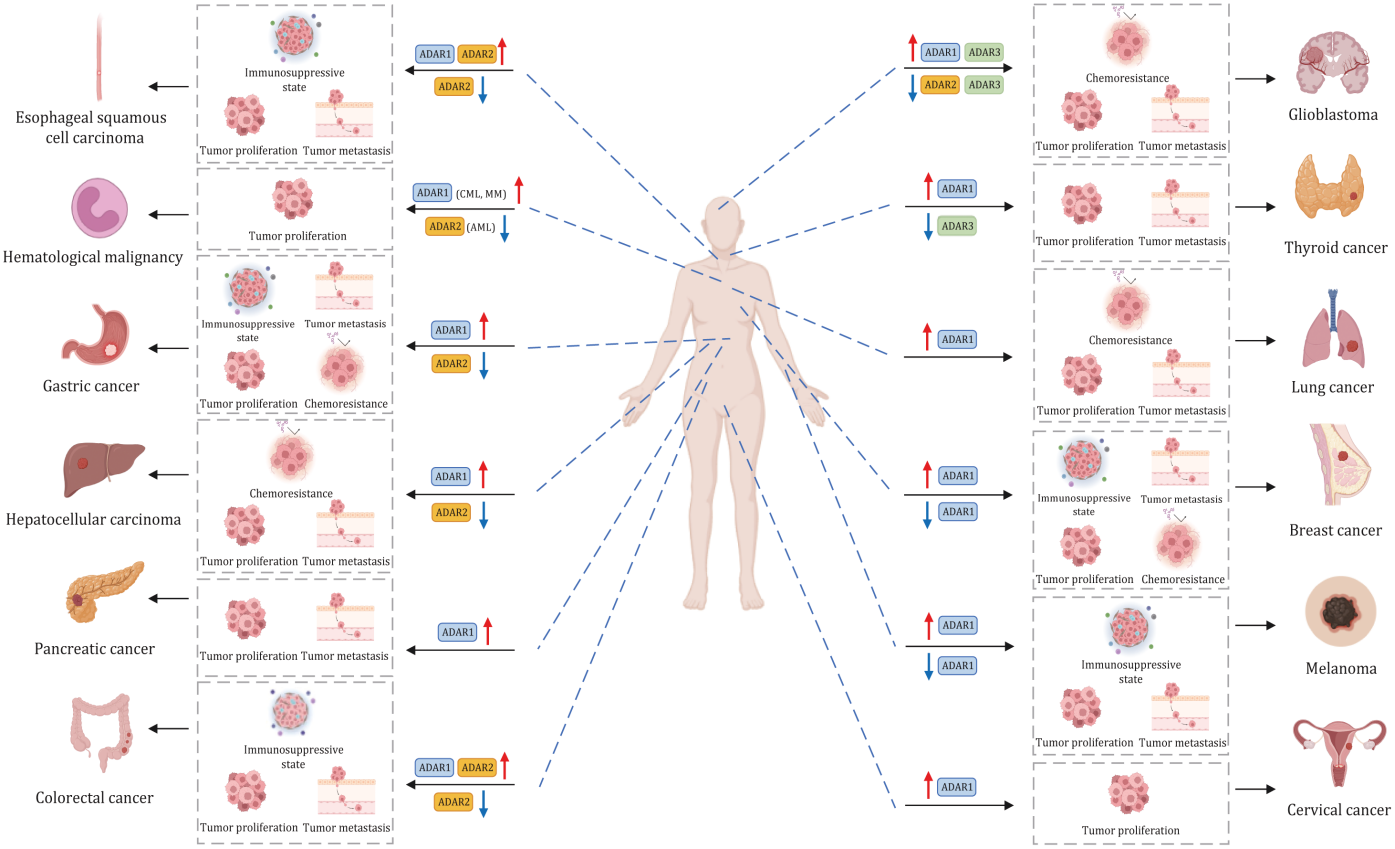
The role of A-to-I regulators in human cancer. ADARs in various human malignancies, and their dual roles in the promotion or suppression of tumorigenesis by modulating cell proliferation, metastasis, or anti-tumor immune response.

**Figure 4.**
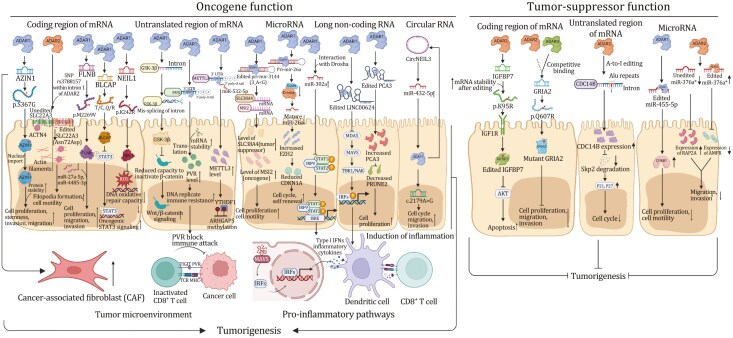
Molecular mechanisms linking dysregulation of RNA A-to-I modification to tumorigenesis. Molecular mechanisms of A-to-I regulators (mainly ADAR1, ADAR2, and ADAR3) in regulating cancer progression through A-to-I modification of mRNA coding region, untranslated mRNA region, microRNA, long noncoding RNA, and circular RNA.

Emerging studies have shown that A-to-I modification plays a significant role in cancer development. RNA adenosine deaminases, especially ADARs, are overexpressed in tumors and they aberrantly catalyze A-to-I modification, eventually influencing the expression of target genes involved in tumorigenesis or modulation of tumor microenvironment (Table 1). Below, we describe the significance of A-to-I editing in a variety of cancers (Figs. 3–4).

**Table 1.** Summary of the function and downstream targets of A-to-I modification in cancer.

Cancer types	A-to-I modifier	A-to-I editing site	Impact on mRNA/protein	Gene(s) involved	Functional implications	Organisms
**A-to-I promoting cancer**						
Acute lymphoblastic leukemia	ADAR1	SNP	The variations are expected to disrupt or enhance interaction with other transcriptional regulatory proteins	rs9616 and rs2229857 genetic variants	The variant at rs9616 and rs2229857 modulates ADAR1 expression and confers a predisposition and relapse risk to ALL	Humans/cell lines
Breast cancer	ADAR1	MiRNA, 3ʹUTR	RNA editing stabilizes DHFR mRNA	miR-25-3p and miR-125a-3p, DHFR	DHFR is posttranscriptionally regulated through ADAR1-mediated RNA editing by editing the miR-25-3p and miR-125a-3p binding sites in the 3’-UTR of DHFR, affecting cell proliferation and sensitivity of BC cells to methotrexate	Humans/cell lines
	ADAR1	MiRNA, 3ʹUTR	Increases METTL3 mRNA and protein levels	METTL3	ADAR1 edits METTL3 mRNA and changes its binding site to miR532-5p, leading to increased METTL3 protein, which further targets ARHGAP5, recognized by YTHDF1 to promote the proliferation, migration, and invasion of BC cells	Humans/cell lines/mice
	ADAR1	LncRNA	Alters LINC00944 expression levels by means of noncanonical functions	LINC00944	Edited lncRNA LINC00944 is immune-related and positively correlates to tumor infiltrating T lymphocytes, the age at diagnosis, tumor size, and poor prognosis	Humans/cell lines
	ADAR1	LncRNA	LINC00624 promotes ADAR1 RNA editing ability by regulating ADAR1 expression	LINC00624	Edited LINC00624 inhibits MHC class I antigen presentation and limits CD8 + Tcell infiltration in the BC microenvironment	Humans/cell lines/mice
	ADAR1	NA	NA	KYNU	Edited KYNU is associated with aggressiveness of BC	Humans/cell lines
	ADAR1	Coding gene	p.M2293V. The editing reduces FLNB activity via disruption of binding partners and consequently of localization	FLNB, miR-27a-5p and miR-4485-3p	Edited FLNB reduces the tumor suppressive activities of the protein, thereby promoting growth and invasion in TNBC. ADAR1-downregulated miRNAs 27a-5p and miR-4485-3p inhibit cell-cycle progression, and that miR-27a-5p also suppresses invasion and promotes IL6/TNF expression in TNBC cells	Cell lines
Cervical cancer	ADAR1	Coding gene	Y/C, Q/R	BLCAP	Edited BLCAP regulates the STAT3 signaling pathway to promote the progression of CC carcinogenesis	Humans/cell lines
Chronic myeloid leukemia	ADAR1	Intron	ADAR1 drives alternative splicing of GSK3β	GSK3β	ADAR1 induces mis-splicing of GSK3β resulting in the renewal of leukemia stem cell	Humans/cell lines/mice
	ADAR1	MiRNA	A-to-G nucleotide changes at + 3 and + 59 editing sites may alter RNA secondary structures at DROSHA/DGCR8 and DICER cleavage sites	Let-7	ADAR1 reduces let-7 levels and enhances leukemic stem cell renewal	Humans/cell lines/mice
	ADAR1	MiRNA	ADAR1 hinders pri-miR-26a biogenesis by preventing DROSHA cleavage	miR-26a	ADAR1 reduces the maturation of miR-26a, which indirectly represses CDKN1A expression via EZH2, hence regulating cell-cycle transit	Humans/cell lines/mice
	ADAR1	3ʹUTR	ADAR1 reduces miR-155 expression	MDM2	ADAR1 edits the 3ʹUTR of MDM2 to prevent targeting by miR-155, leading to increase MDM2 levels and inhibit the activation of p53, thereby promoting the progression of CML	Humans/cell lines/mice
Colorectal cancer	ADAR1	Coding gene	S/G	AZIN1	Edited AZIN1 enhances stemness and appears to drive the metastatic process	Humans/cell lines/mice
	ADAR2	Coding gene	Q/R in the fifth amino acid of the BLCAP protein	BLCAP	Edited BLCAP facilitates the transition from G1 to S phase of the cell cycle through the loss of repressive effect on Rb1, leading to the increased cell proliferation and reduced apoptosis	Humans/cell lines/mice
	ADAR1	3ʹ-UTR	Increases the RNA stability	PVR	ADAR-mediated RNA editing may enhance tumor- and immune-related gene activities and pathways in CRC by upregulating PVR expression.	Human/cell lines
	ADAR1	Coding gene	S/G	AZIN1	Edited AZIN1 enhances the invasive potential of CAFs within the TME	Humans/cell lines
Esophageal squamous cell carcinoma	ADAR1	Coding gene	p.S367G	AZIN1	Edited AZIN1 transcript is more abundant in tumors, resulting in “gain of function” phenotypes during ESCC progression	Humans/cell lines/mice
	ADAR1	NA	NA	SOX2	Oncogenic Sox2 activates endogenous retroviruses, inducing expression of dsRNA and dependence on the ADAR1	Human/cell lines/mice
	ADAR2	Coding gene (c.261A > G)	p.N72D	SLC22A3	Deregulation of SLC22A3 facilitates cell invasion and filopodia formation by reducing its direct association with ACTN4, leading to the increased actin-binding activity of ACTN4 in normal esophageal cells	Humans/cell lines/mice
Gastric cancer	ADAR1	NA	NA	mTOR	ADAR1 activates mTOR/p70S6K/S6 ribosomal protein signaling axis to regulate protein translation, cell proliferation, and autophagy	Humans/cell lines/mice
	ADAR1	MiRNA	ADAR1 mediates its effect via the interaction with DROSHA or by regulating the transcription of miR-302/367 cluster	miR-302a-3p and IRF9	ADAR1 regulates IFN signaling through the suppression of STAT1 and IRF9 via miR-302a	Cell lines
	ADAR1	3′UTR	A-to-I editing on 3′UTR of SCD1 enhances binding of KHDRBS1, thereby increasing SCD1 mRNA stability	SCD1	ADAR1/SCD1 axis governs chemoresistance by enhancing lipid droplet formation to neutralize ER stress induced by chemotherapy	Humans/cell lines/mice
	ADAR1	CircRNA	ADAR1 has the ability to reduce the abundance of hsa_circ_0004872 in GC cells	hsa_circ_0004872 and miR-224	ADAR1 inhibits the expression of hsa_circ_0004872, which led to the upregulation of miR-224. Smad4, the target of miR-224, could further influence hsa_circ_0004872 levels by binding directly to the promoter region of ADAR1 to inhibit ADAR1 expression	Humans/cell lines/mice
Glioblastoma	ADAR1	NA	ADAR1 binds and stabilizes CDK2 transcript	CDK2	ADAR1 stabilizes CDK2 a key player in cancer cell-cycle progression, so promoting GBM proliferation *in vitro* and most importantly *in vivo*	Humans/cell lines/mice
	ADAR3	Coding gene	Q/R, ADAR3 inhibits editing of the Q/R site by binding to the GRIA2 pre-mRNA	GRIA2	ADAR3 expression contributes to the relative level of GRIA2 editing in tumors from patients suffering from glioblastoma	Humans/cell lines
	ADAR3	NA	ADAR3 leads to increased phosphorylation and translocation of NF-κB into the nucleus	NF-κB	ADAR3 promotes NF-κB activation and a gene expression program that provides a growth advantage to glioblastoma cells	Cell lines
Hepatocellular carcinoma	ADAR1	Coding gene	p.M2269V	FLNB	Hyper-editing of FLNB (filamin B, β) is closely linked to HCC pathogenesis	Humans/cell lines/mice
	ADAR1	CircRNA	A-to-I editing occurs near the location of reverse complementary matches	CircARSP91	CircARSP91 downregulated by AR in an ADAR1-dependent manner, could inhibit HCC tumor growth both *in vitro* and *in vivo*	Humans/cell lines/mice
	ADAR1	NA	NA	ITGA2	ADAR1 enhances HCC metastasis by promoting tumor cells adhering to ECM via increasing ITGA2 expression	Humans/cell lines/mice
	ADAR1	MiRNA	ED_miR-3144-3p(3_A < G)	miR-3144-3p	ADAR1 augments oncogenic MSI2 effects by editing miR-3144-3p and that the resultant ED_miR-3144-3p(3_A < G) simultaneously suppresses tumor suppressor SLC38A4 expression, contributing to hepatocellular carcinogenesis	Humans/cell lines/mice
	ADAR1	Coding gene	p.S367G. This substitution is predicted to cause a conformational change, resulting in a translocation from the cytoplasm to the nucleus	AZIN1	Edited AZIN1 has a stronger affinity to antizyme and promotes cell proliferation through the neutralization of antizyme-mediated degradation of ODC and CCND1	Humans/cell lines/mice
Lung cancer	ADAR1	Coding gene and MiRNA	p.K242R. ADAR1 binds and edits on the neighboring pri-miR-381 sequence	NEIL1 and miR-381	ADAR1 promotes tumor growth and mediates the regulation of A-to-I editing in both coding (NEIL1) and noncoding (miR-381) RNA transcripts	Humans/cell lines/mice
	ADAR1	NA	NA	CX3CR1	ADAR1 deficiency increases the sensitivity of NSCLC/AR cells to Anlotinib by downregulating CX3CL1	Cell lines/mice
	ADAR1	3′UTR	ADAR1 mainly binds to the segment chr17: 82034049-82034204 of the 3ʹ-UTR in RAC3 mRNA	DDX1	DDX1 mediates chemosensitivity to cisplatin via the ADAR1/RAC3 axis and promotes cancer progression	Humans/cell lines/mice
	ADAR1	Intron	Targets a specific region within an intron on chromosome 8 (position 141,702,274) in the FAK transcript, leading to enhanced stability of FAK mRNA	FAK	ADAR1 increases FAK protein abundance posttranscriptionally by binding to the FAK transcript and modifying a specific intronic site, resulting in the increased stabilization of FAK mRNA	Humans/cell lines
Melanoma	ADAR1	MiRNA	ADAR1p150 increases and the expression level of miRNA-149*	miRNA-149*	ADAR1 forms a complex with Dicer and enhance the function of miRNA-149*, thereby promoting proliferation of melanoma cells and inhibited cell apoptosis	Humans/cell lines
Mesothelioma	ADAR2	3′UTR	ADAR2 maintains the level of DHFR	DHFR, FPGS	ADAR2 deficiency leads to upregulation of type 1 IFN response and sensitizes mesothelioma cells to pemetrexed via ADAR2/DHFR, FPGS pathway	Humans/cell lines
Multiple myeloma	ADAR1	Coding gene	p.R701G	GLI1	Edited GL1 activates the Hedgehog pathway and promotes malignant self-renewal of multiple myeloma *in vivo* and immunomodulatory drug resistance *in vitro*	Humans/cell lines/mice
	ADAR1	Coding gene (c.726A > G)	p.K242R	NEIL1	Recoded NEIL1 protein demonstrated deficient oxidative damage repair capacity and loss-of-function characteristics.	Humans/cell lines/mice
Pancreatic cancer	ADAR1	Coding gene	ADAR1 suppresses the phosphorylation of AKT at Ser-473 sites	AKT, c-Myc	ADAR1 stabilizes c-Myc via AKT signaling, which contributes to cancer cell resistance to BET inhibitors in PC cells	Humans/cell lines/mice
	ADAR1	Coding gene	A-to-I editing occurs in exon 12 of GLI1 mRNA, specifically at nucleotide position chr12:57864624	GLI1, circNEIL3	CircNEIL3 promotes the proliferation and metastasis of PC via the circNEIL3/miR-432-5p/ADAR1/GLI1/cell cycle and EMT axis and that its expression is negatively regulated by ADAR1 via a feedback loop	Humans/cell lines/mice
Prostate cancer	ADAR1	LncRNA	ADAR1 interacts with PRUNE2/PCA3 dsRNA and controls the levels of PRUNE2	PCA3 and PRUNE2	PCA3 as a transdominant negative oncogene that inhibits the unidentified tumor suppressor gene PRUNE2 at the RNA level through an ADAR-mediated mechanism	Humans/cell lines/mice
Thyroid cancer	ADAR1	MiRNA	The editing hotspot in miR-200b is located in its seed region	miR-200b	The impaired ability of edited miR-200b to inhibit ZEB1 could promote motility and invasion in TC cells	Humans/cell lines/mice
	ADAR1	Coding gene (c.308A > G)	p.Q103R	CDK13	Edited CDK13-Q103R stimulates a stronger cell proliferation and migration phenotype than WT CDK1	Humans/cell lines
**A-to-I suppressing cancer**						
						
Acute myeloid leukemia	ADAR2	Coding gene	COPA(p.I164V) COG3(p.I635V)	COPA and COG3	ADAR2 suppresses leukemogenesis specifically in t(8;21) and inv16 AML cells by the RUNX1-ETO AE9a fusion protein, targeting COPA and COG3 to inhibit clonogenic growth of human t(8;21) AML cells	Humans/cell lines/mice
Breast cancer	ADAR1	Coding gene	p.I342M	GABRA3	Edited Gabra3 suppresses BC cell invasion and metastasis	Cell lines/mice
Colorectal cancer	ADAR2	MiRNA	ADAR2 regulates the secretion of miR-200s or influences the levels of RBPs or other components of the miRNA secretion machinery	miR-200s	PKCζ-phosphorylated ADAR2 promotes the accumulation of miR-200s and loss of the PKCζ/ADAR2 axis results in EMT and increased liver metastases	Humans/cell lines/mice
Esophageal squamous cell carcinoma	ADAR2	Coding gene (c.284A > G)	p.K95R	IGFBP7	ADAR2 edits and stabilizes IGFBP7 to suppresses tumor growth and induces apoptosis in ESCC	Humans/cell lines/mice
Gastric cancer	ADAR2	Coding gene	p.H241R	PODXL	Edited PODXL at codon 241 (His to Arg) confers a loss-of-function phenotype that neutralizes the tumorigenic ability of the unedited PODXL	Humans/cell lines/mice
Glioblastoma	ADAR2	MiRNA	ADAR2 reduces the levels of miR-221, -222 and -21 by preventing their precursors from maturing.	miR-222/221 and miR-21	ADAR2 edits miR-222/221 and miR-21 precursors and decreases the expression of the corresponding mature onco-miRNAs *in vivo* and *in vitro*, with significant effects on cell proliferation and migration	Humans/cell lines/mice
	ADAR2	Intron	Edited CDC14B pre-mRNA enhances its expression and therefore reduces the skp2 target protein	CDC14B	ADAR2 inhibits astrocytoma growth by increasing the level of CDC14B, which in turn affects the Skp2/p21-p27 pathway	Humans/cell lines/mice
Glioma	ADAR2	MiRNA	The A-to-I editing of miR-378a occurs within the seed region	miR-376a*	Unedited miR-376a* decreases RAP2A, but a modified miR-376a* causes an accumulation of AMFR, collectively leading to increased migration and invasiveness of glioma cells	Humans/cell lines/mice
	ADAR3	Coding gene	p.Q607R	GRIA2	Low expression of ADAR3 may induce unedited GRIA2 transcripts level that can promote cell migration and tumor invasion	Humans/cell lines
Hepatocellular carcinoma	ADAR2	SNP	The rs2253763 C-to-T change in ADAR2 3′UTR reduces its interaction with miR-542-3p and allele-specifically elevates ADAR2 levels	rs2253763 genetic variants	The rs2253763 SNP in ADAR2 3’-UTR could disturb miR- 542-3p binding, leading to dysregulated production of ADAR2 in an allelic way and ADAR2 could enhance oxaliplatin sensitivity	Humans/cell lines
	ADAR2	Coding gene	p.I164V	COPA	Hypo-editing of COPA (coatomer protein complex, subunit α) is closely linked to HCC pathogenesis	Humans/cell lines/mice
Melanoma	ADAR1	MiRNA	ADAR1 regulates biogenesis of miRNAs directly by potentially affecting Drosha complex and indirectly by regulating Dicer via let-7	miR-17, miR-432	Overexpression of miR-17-5p and miR-432 mediates the loss of ADAR1 in cancer cells and subsequently increases the malignant features	Humans/cell lines/mice
	ADAR1	MiRNA	The reduction of ADAR1 results in aberrant miRNA processing, as well as alterations in the overall levels of mature miR-455-5p	miR-455-5p	Edited miR-455-5p inhibits melanoma metastasis through promotion of the tumour suppressor gene CPEB1	Cell lines/mice
	ADAR1	MiRNA	Edited miR-22 regulates the stability of the mRNA of ITGB3	ITGB3, miR-22 and PAX6	ADAR1 regulates ITGB3 expression via miR-22 and PAX6 transcription factor both at the posttranscriptional and transcriptional levels, respectively	Humans/cell lines
	ADAR1	MiRNA	Reduced miR-30a/d lead to a significant increase in ITGB3 expression in all three melanoma lines at the mRNA level	ITGB3, miR-30a and miR-30d	ADAR1 regulates ITGB3 by pointing on miR-30a and miR-30d as ADAR1-controlled microRNAs, which play a direct role in the posttranscriptional expression control of ITGB3 and of the invasive melanoma cell phenotype	Humans/cell lines/mice
	ADAR1	MiRNA	ADAR1 influences the transcription of miR222 precursors	miR-222	ADAR1 regulates the biogenesis of miR222 and thereby ICAM1 expression, which consequently affects melanoma immune resistance	Humans/cell lines
	ADAR1	MiRNA	The A-to-I editing of miR-378a-3p is non-canonical and it occurs outside the seed region	miR-378a-3p	Edited miR-378a-3p binds to the 3ʹ-UTR of the PARVA oncogene and inhibits its expression, thus preventing the progression of melanoma towards the malignant phenotype	Cell lines/mice
Thyroid cancer	ADAR3	SNP	The ADAR3 rs904957 polymorphism, which involves a thymine-to-cytosine (T-to-C) change in the 3′UTR, increases the binding affinity of miR-1180-3p and suppresses ADAR3	rs904957 genetic variants	ADAR3 rs904957 genetic polymorphism located in the gene 3’-UTR results in allelic downregulation of ADAR3 expression and miR-1180-3p posttranscriptionally suppresses ADARB2 expression	Humans/cell lines

NA: not available.

### A-to-I editing of mRNA in tumorigenesis

ADAR1 is overexpressed in many cancers and primarily functions as an oncogene (Qin et al., 2014; Takeda et al., 2019) by promoting cell proliferation and migration (Dou et al., 2016; Sun et al., 2020). Multiple studies have demonstrated that hyper A-to-I editing of AZIN1, modulated by ADAR1, is associated with the tumorigenesis of colorectal cancer (CRC), endometrial cancer (Nakamura et al., 2022), gastric cancer (GC) (Wang et al., 2023a), liver cancer (Shibata et al., 2023), and esophageal squamous cell carcinoma (ESCC) (Qin et al., 2014), and that recoding of AZIN is correlated with worse prognosis. Mechanistically, A-to-I editing of AZIN1 transcripts led to a serine-to-glycine substitution at residue 367, which is localized to β-strand 15 and is predicted to cause a conformational change that promotes AZIN cytoplasmic-to-nuclear translocation and also increases its protein stability (Chen et al., 2013). AZIN1 functions as an oncogenic factor that enhances cell proliferation, invasion, and migration capabilities; and cancer stemness characteristics (Chen et al., 2013; Shigeyasu et al., 2018), thereby conferring gain-of-function phenotypes with augmented tumor-initiating potential and aggressive phenotypes. Beyond cancer cells, increased expression of AZIN1 through A-to-I editing enhances invasive potential of cancer-associated fibroblasts (CAFs) within the tumor microenvironment in colon, and is an important predictor of tumor invasiveness in CRC (Takeda et al., 2019). Alterative targets of ADAR1-driven recoding with gain-of-function have been reported. ADAR1 enhances the function of GLI1 through A-to-I editing that leads to a change from arginine to glycine at Position 701, which boosted GLI1 transcriptional activity and therefore pro-tumorigenic phenotypes in pancreatic ductal adenocarcinoma (Shen et al., 2021). In thyroid cancer (TC), ADAR1-mediated modification of CDK13 leads to a change from glutamine to arginine at Position 103. Edited CDK13 protein was found to be enriched in the nucleolus and it conferred enhanced cell proliferation and migration properties to TC cells (Ramírez-Moya et al., 2021).

Nevertheless, more frequently ADAR1-mediated A-to-I recoding events inactivate tumor suppressors to induce tumor progression. ADAR1 is a critical oncogene for triple-negative breast cancer (TNBC). Substitution of methionine by valine at residue 2,269 in filamin B (FLNB) due to recoding by ADAR1 abrogates tumor suppressive activities of the protein, leading to cell-cycle progression and invasion (Baker et al., 2022). In cervical cancer, ADAR1 modified the BLCAP transcripts at two sites (tyrosine-to-cysteine and glutamine-to-arginine) in its coding region-tyrosine-X-X-glutamine (YXXQ) motif that binds to Src-homology 2 domain of signal transducer and activator of transcription 3 (STAT3) and inhibits its phosphorylation. A-to-I recoding of BLCAP prevents its interaction to STAT3, leading to the activation of STAT3 signaling and cancer progression (Chen et al., 2017a). ADAR1 also functions as an oncogenic factor in multiple myeloma (MM). Mechanistically, NEIL1 (required for base excision repair) is a target of ADAR1 A-to-I editing. The editing of NEIL1 on exon 6 leads to lysine-to-arginine substitution at Position 242, and recoded NEIL1 protein demonstrates defective oxidative damage repair and loss-of-function characteristics, which consequently promotes the acquisition of sporadic mutations in MM (Teoh et al., 2018). In contrast to the prevailing notion that ADAR1-mediated recoding is largely pro-tumorigenic in nature, one report demonstrated that ADAR1p110-mediated A-to-I recoding of GABRA3 (isoleucine-to-methionine at Position 342) repressed GABRA3-mediated AKT signaling, leading to reduced cell migration and invasion in BC (Gumireddy et al., 2016).

Besides recoding, ADAR1-mediated A-to-I editing at 3ʹ-UTR contributes to tumorigenic phenotypes. ADAR1 A-to-I modified 3ʹ-UTR of MDM2 and prevents its targeting by miR-155. Increased MDM2 in turn compromises transcriptional activation of p53 and promotes CML development (Jiang et al., 2019). In CRC, ADAR-mediated RNA editing at 3ʹ-UTR of PVR could upregulate its expression by increasing the RNA stability, leading to tumor- and immune-related gene functions and pathways in CRC (Qian et al., 2024). ADAR1-catalyzed A-to-I RNA editing of 3ʹ-UTR of Rho GTPase activating protein 26 (ARHGAP26) mRNA abolished its pairing to miR-30b-3p and miR-573, allowing increased translation and protein expression of this oncogene in BC (Wang et al., 2013b). ADAR1 has also been shown to modify 3ʹ-UTR region of methyltransferase-like 3 (METTL3), an *N*^6^-methyladenosine (m^6^A) writer, and abrogates its interaction with miR-532-5p, resulting in increased METTL3 protein expression in BC cells (Li et al., 2022c). This, in turn, promotes m^6^A modification and translation of ARHGAP5, a driver of tumor progression and metastasis. Moreover, METTL3-driven m^6^A modification reciprocally boosts ADAR1 mRNA and protein expression (Tassinari et al., 2021), implying positive feed-forward circuitry between A-to-I editing and m^6^A modification in promoting tumorigenesis. Recent studies identified an A-to-I editing independent mechanism of ADAR1 involving 3ʹ-UTR. Phosphorylated ADAR1 (ADAR1p110) in the cytosol binds to 3ʹ-UTR of several anti-apoptotic genes, which prevents their binding to Staufen1 and subsequent mRNA degradation, thereby promoting cancer cell survival (Boulay et al., 2014; Crawford Parks et al., 2017; Gong and Maquat, 2011; Sakurai et al., 2017; Xu et al., 2015).

The role of intronic A-to-I editing in tumorigenesis remains understudied. One study has reported that A-to-I editing of focal adhesion kinase (FAK) at specific intron sites on chr8,141,702,274 increases the stability of FAK mRNA and expression of FAK protein to promote mesenchymal traits, migration and invasion of lung adenocarcinoma (LUAD) (Amin et al., 2017). In CML, A-to-I editing within the introns of glycogen synthase kinase 3β (GSK-3β) by ADAR1 caused the mis-splicing of GSK-3β with reduced capacity to inactivate β-catenin, thereby facilitating LSCs self-renewal via the activation of β-catenin signaling (Abrahamsson et al., 2009; Jiang et al., 2013). ADAR1-driven A-to-I editing thus has wide ranging pro-tumorigenic effects in multiple cancer types.

In contrast to ADAR1, ADAR2-mediated A-to-I editing is frequently associated with tumor suppression. In liver cancer, ADAR1 overexpression and ADAR2 downregulation predict poor prognosis and increased risk of postoperative recurrence (Chan et al., 2014), an effect attributed to the differential selectivity in mRNA targeting by ADAR1/2. Indeed, several tumor suppressors have been identified as targets of ADAR2. For instance, ADAR2 targets intronic Alu elements of phosphatase cell division cycle 14B (CDC14B), an upstream regulator of S-phase kinase-associated protein 2 (Skp2)/p21/p27 pathway, in glioblastoma (GBM) (Galeano et al., 2013). ADAR2-mediated A-to-I editing on CDC14B pre-mRNA increases its expression, with a consequent reduction of Skp2 and the induction of p21/p27-mediated cell-cycle arrest (Galeano et al., 2013). In core binding factor acute myeloid leukemia (AML), which is defined by cytogenetic abnormalities either due to translocation (8;21) (q22; q22.1) or due to inversion (16) (p13.1; q22), ADAR2 is down-regulated due to the expression of RUNX1-ETO additional exon 9a fusion protein that exerts a dominant negative effect on ADAR2 transcription (Darwish et al., 2023; Guo et al., 2023). This led to downregulation of ADAR2 targets coatomer subunit α (COPA) (isoleucine-to-valine substitution at reside 164) and the component of oligomeric Golgi complex 3 (COG3) (isoleucine-to-valine substitution at residue 635), both of which inhibit clonogenic growth. In ESCC, ADAR2 also had tumor suppressive function. ADAR2 promotes asparagine-to-aspartate change at residue 72 of SLC22A3, a novel metastasis suppressor in ESCC (Fu et al., 2017). Downregulation of SLC22A3 facilitates cell invasion and filopodia formation by abrogating the binding between SLC22A3 and α-actinin-4 (ACTN4). ADAR2 also modifies the insulin-like growth factor binding protein 7 (IGFBP7) mRNA and stabilizes its protein by altering protease recognition site of matriptase (lysine-to-arginine at residue 95), which is essential for IGFBP7-induced apoptosis and the inhibition of Akt signaling in ESCC (Chen et al., 2017b). In gastric cancer, ADAR2-induced recoded podocalyxin-like (PODXL) (histidine-to-arginine substitution at residue 241) protein was found to suppress tumorigenesis by neutralizing the tumorigenic capacity of wild-type PODXL. In this scenario, ADAR1 and ADAR2 were observed to exert mutual interference on the binding and A-to-I modification of PODXL, whereby overexpression of ADAR1 impairs the tumor suppressive function of ADAR2 (Chan et al., 2016; Thomas, 2016). On the contrary, other studies have described pro-tumorigenic function of ADAR2. In CRC, A-to-I editing of BLCAP by ADAR2 leads to a substitution from glutamine to proline at residue 5, accelerating degradation of BLCAP via the ubiquitination-proteasome pathway (Han et al., 2022). As BLCAP interacts with retinoblastoma 1 (Rb1) to prevent its inactivation, downregulation of BLCAP by ADAR2 accelerates G_1_-S cell-cycle transition, increases cell growth, and inhibits apoptosis. Another study also demonstrated oncogenic function of ADAR2 in malignant pleural mesothelioma (PM), albeit such effects are independent of RNA editing function (Sakata et al., 2020).

Compared to ADAR1/2, few studies have shed light on the potential role(s) of ADAR3 in cancer. As it is devoid of A-to-I editing functionality, ADAR3 mediates its effect on cancer by inhibiting RNA editing, mostly notably that of ADAR2-mediated glutamate receptor ionotropic AMPA2 (GRIA2) A-to-I editing (glutamine-to-arginine substitution at residue 607) in glioma and glioblastoma (Oakes et al., 2017; Zhang et al., 2018). More work is required to uncover the exact role of ADAR3 in cancer. In summary, current evidence supports a largely oncogenic function of ADAR1-driven A-to-I editing in cancer, whereas ADAR2 might possesses tumor suppressive or pro-tumorigenic function in a cancer type-specific and context-dependent manner.

### A-to-I editing of noncoding RNA in tumorigenesis

A-to-I editing primarily modulates targeting and maturation of noncoding RNAs, thereby affecting their roles in cancers. ADAR1 also promotes pre-miRNA cleavage by Dicer and loading of mature miRNA into RISC independently of its RNA editing function (Ota et al., 2013). A number of microRNAs have been shown to be edited by ADARs. A-to-I editing of miR-3144-3p (3_A < G) by ADAR1 in liver cancer drastically shifts the target specificity of this miRNA, with the defective target of its canonical mRNA target Musashi RBP 2 (MSI2, an oncogene), whereas generating gain-of-function binding to SLC38A4 mRNA (a tumor suppressor). This led to overexpression of MSI2 concomitant with the downregulation of SLC38A4, thereby promoting tumorigenesis (Kim et al., 2023). In CML progenitors, ADAR1-induced hyper-editing of compromises maturation of pri-miR-26a by preventing its cleavage by DROSHA, a tumor suppressor miRNA that inhibits cell-cycle progression. Down-regulated miR-26a therefore accelerated cell cycle and self-renewal (Jiang et al., 2019). In LUAD, ADAR1 mediates A-to-I editing of miR-381, a microRNA implicated in stemness, chemoresistance, and other cancer-relevant pathways (Anadón et al., 2016). In TC, the hyper-editing of miR-200b by ADAR1 attenuated the capacity of miR200b to bind to ZEB1, a major transcription factor driving epithelial–mesenchymal transition (EMT). Knockdown of ADAR1 thus suppressed ZEB1, EMT, and aggressivity of TC cells (Ramírez-Moya et al., 2020).

On the other hand, several studies have shown that ADAR1 editing of miRNAs contributes to an antimetastatic effect in melanoma. ADAR1 is frequently down-regulated in metastatic melanoma. ADAR1 silencing increased the expression of non-edited miR-455-5p, which promotes the metastasis through inhibition of tumor suppressor gene CPEB1 (Shoshan et al., 2015). In a similar vein, miR-378a-3p is edited by ADAR1 in nonmetastatic but not in metastatic melanoma cells, and the modified form of miR-378a-3p preferentially binds to 3ʹ-UTR of the alpha-parvin (PARVA) oncogene and suppresses its expression, thereby preventing progression of melanoma towards malignant phenotypes (Velazquez-Torres et al., 2018). Moreover, ADAR1 silencing impairs cell invasiveness in melanoma by downregulating β3-integrin at both posttranscriptional and transcriptional levels via paired box 6 (PAX6) and miR-22/miR-30a/d, respectively (Nemlich et al., 2018, 2020). Additionally, ADAR1 modulates miRNA processing in a RNA editing-independent manner by regulating Dicer, an enzyme that cleaves precursor miRNA, thereby promoting the biosynthesis and function of miRNA-149* and negatively correlated with the GSK3a expression in melanoma (Nemlich et al., 2013; Yujie Ding et al., 2020). These findings suggest that ADAR1 editing of miRNA might exert discordant effect on tumorigenesis in a cancer type- and stage-specific dependent manner.

Similar to that its role in editing mRNA, ADAR2-mediated miRNA editing has been linked with tumor suppressive function(s). In CRC, ADAR2 is directly phosphorylated by protein kinase C ζ (PKCζ), which promotes ADAR2 editing activity and is necessary to maintain miR-200, an EMT repressor. Depletion of ADAR2 led to decreased miR-200, EMT and liver metastases (Shelton et al., 2018). miR-376a*, one of the mature miRNAs from miR-376 cluster, has been shown to facilitate glioma growth, migration and invasion in unedited form. ADAR2-driven editing of miR-376a* inhibited oncogenic characteristics of this miRNA (Choudhury et al., 2012). Mechanistically, ADAR2-editing shifted the target specificities of miR-376a* from RAP2A (member of the RAS oncogene family) to autocrine motility factor receptor (AMFR), resulting in increased RAP2A together with downregulation of AMFR to mediate pro-tumorigenic and pro-metastatic effects. Global profiling of miRNA in GBM cells expressing wildtype or catalytic-dead ADAR2 revealed that ADAR2 loss-of-function induced the expression of ~90 miRNA, the majority of which are onco-miRNAs. ADAR2 editing of miR222/221 and miR-21 precursors, for example, reduces expression of mature onco-miRNAs *in vitro* and *in vivo*, resulting in impaired cell proliferation and metastasis of GBM (Tomaselli et al., 2015). Hence, ADAR2 editing of miRNA likely exerts tumor suppressive function in cancer.

Beyond its wide-ranging effects on miRNA expression and specificity, ADAR1 functions as a repressor of circRNA production, which is a consequence of A-to-I editing near reverse complementary matches, a structural element considered essential for circRNA synthesis (Ivanov et al., 2015). In hepatocellular carcinoma (HCC), Shi et al. (2017) demonstrated that androgen receptor (AR) transcriptionally activates ADARp110 isoform, which then downregulates circARSP91 (hsa_circ_0085154), a tumor suppressive circRNA. AR-activated ADAR1 RNA editing might thus contribute to sexual disparity in HCC. Several circRNAs, such as hsa_circ_0004872 (Ma et al., 2020) and circNEIL3 (Shen et al., 2021), have been shown to reciprocally regulate ADAR1 expression in cancer, suggesting complex interactive circuity between circRNAs and ADAR1-mediated editing in driving tumorigenesis.

## Role of A-to-I in cancer therapy

### A-to-I editing in chemotherapy and targeted therapy response

**Figure 5.**
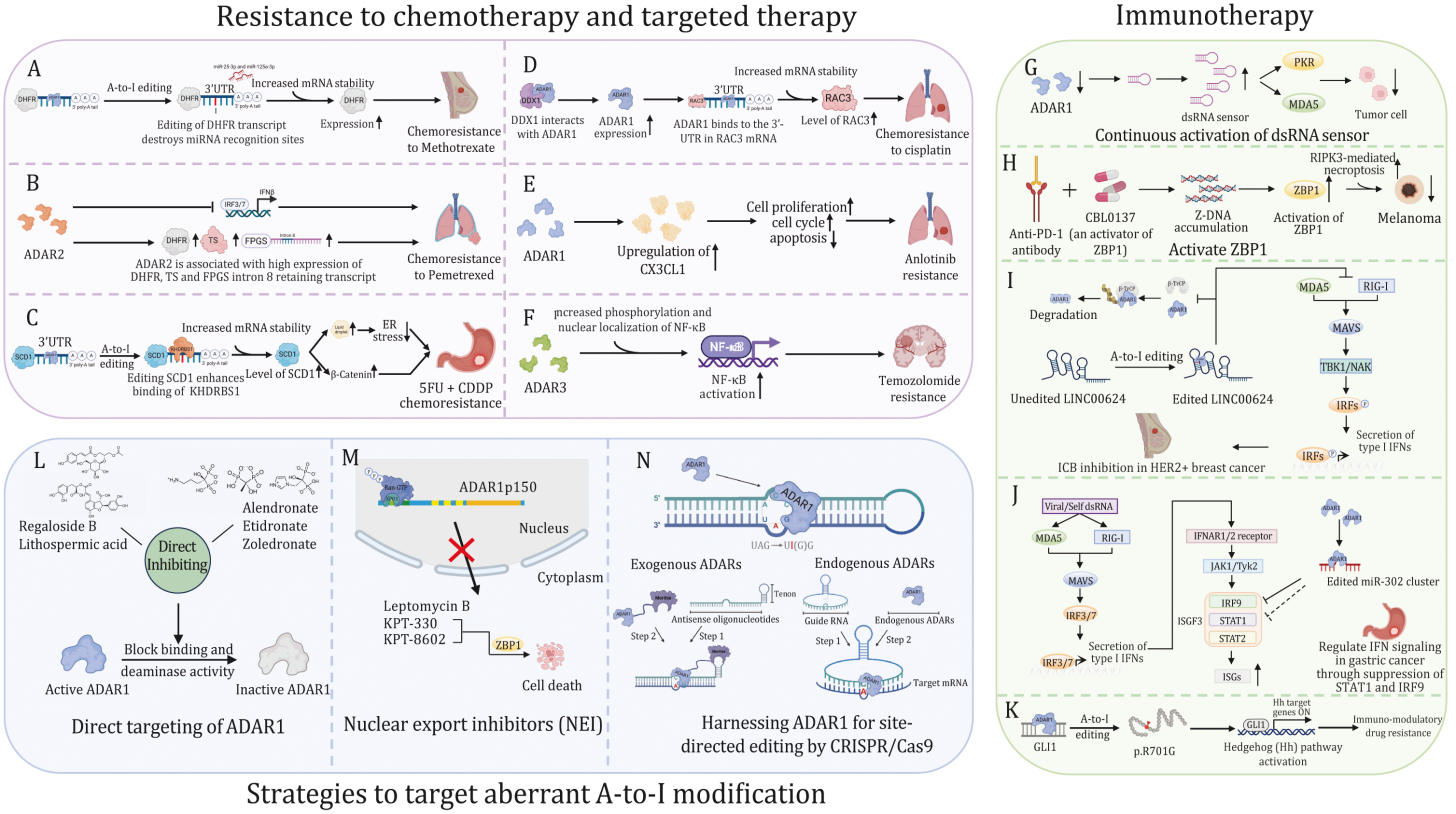
Therapeutic approaches targeting A-to-I RNA editing in cancer. (A) ADAR1 positively regulates the expression of DHFR by editing the miR-25-3p and miR-125a-3p binding sites in the 3ʹ-UTR of DHFR, enhancing resistance to methotrexate. (B) ADAR2 drive chemo-resistance to pemetrexed in mesothelioma cells by promoting DHFR mRNA expression and splicing of active folylpolyglutamate synthetase. (C) ADAR1–KHDRBS1–SCD1 axis drives CRC chemoresistance by enhancing lipid droplet formation. (D) DDX1 mediates cisplatin chemoresistance via an ADAR1/RAC3 axis. (E) ADAR1 enhances Alu-dependent editing and transcriptional activity of GLI1 and promotes immunomodulatory drug resistance. (F) ADAR1 desensitizes NSCLC/AR cells to Anlotinib by downregulating CX3CL1. (G) ADAR3 promotes NF-κB activation and impacts resistance to temozolomide in glioblastoma cells. (H) Anti-cancer epigenetic inhibitors promote the transcription of repetitive sequences that form dsRNAs and activate the dsRNA sensor pathway. (I) Z-DNA inducer CBL0137 induces the formation of Z-DNA and activates ZBP1. (J) Edited LINC00624 stabilized ADAR1 and further suppressed the innate immune response caused by the IFN response in BC cells. (K) ADAR1 regulates IFN signaling in gastric cancer through the suppression of STAT1 and IRF9 via miR-302a. (L) Multiple potential inhibitors direct target the binding site and deaminase activity of ADAR1. (M) NEIs such as KPT-330, KPT-8602, and leptomycin B sequester ADAR1p150 in the nucleus. (N) Site-directed RNA editing by harnessing ADARs.

Chemotherapy is still the mainstays of the treatment of cancers; however, chemoresistance is frequent cause of poor prognosis. Studies have demonstrated that mRNA A-to-I editing adversely impacts chemotherapy in cancer. For instance, ADAR1 controls the expression of dihydrofolate reductase (DHFR), the molecular target of antifolate drug methotrexate, via A-to-I editing of miR-25-3p and miR-125a-3p binding sites in DHFR 3ʹ-UTR (Fig. 5A) (Nakano et al., 2017). In BRCA1-associated protein 1 (BAP1) wild-type mesothelioma, ADAR2 up-regulation enhanced the A-to-I editing of transcripts and 3ʹ-UTR. ADAR2 promotes DHFR expression and splicing of active folylpolyglutamate synthetase (FPGS), both of which drive resistance to antifolates. As a consequence, the depletion of ADAR2 sensitized mesothelioma cells to pemetrexed, a first-line antifolate chemotherapy for mesothelioma (Fig. 5B) (Hariharan et al., 2022). A recent study demonstrated that ADAR1-mediated A-to-I editing on the 3ʹ-UTR of stearoyl-CoA desaturase (SCD1) augments SCD1 mRNA stability in gastric cancer cells (Wong et al., 2023). Increased SCD1 promotes lipid droplet formation to alleviate ER stress and boost cancer stemness, leading to gastric cancer chemoresistance (Fig. 5C). Consistently, Yang et al. (2023) showed that the stabilization of ADAR1 protein by DEAD-box helicase 1 (DDX1) promotes malignant phenotypes and cisplatin resistance (Fig. 5D). These studies suggest ADAR1/2-mediated A-to-I editing as a potential therapeutic target in combination with chemotherapy.

ADAR1 is overexpressed in Anlotinib-resistant non-small cell lung cancer (NSCLC/AR). Knockdown of ADAR1 decreases expression of C-X3-C motif chemokine ligand 1 (CX3CL1) in NCI-H1975/AR and A549/AR cells after Anlotinib treatment. Exogenous CX3CL1 reverses the effect of ADAR1 deficiency on increased NSCLC/AR cell sensitivity to Anlotinib (Wu et al., 2022), implying that ADAR1–CX3CL1 axis promotes Anlotinib resistance (Fig. 5E). ADAR3, although devoid of A-to-I editing activity, could also confer resistance to alkylating agent temozolomide in GBM cells by activating nuclear factor-kappa B (NF-κB) (Raghava Kurup et al., 2022). Hence, ADAR1 overexpression in cancer could elicit resistance to drugs with differential molecular mechanisms of action (Fig. 5F).

### A-to-I editing in immunotherapy response

Immune checkpoint blockade (ICB) therapy based on antibody-mediated blockade of key checkpoint molecules, such as cytotoxic T lymphocyte-associated protein 4 (CTLA-4) and programmed cell death protein 1 (PD-1), have achieved clinical success in some cancers. Nevertheless, many cancers are refractory to ICB therapy, prompting the need for potential adjuvants for improving its efficacy. One promising strategy to improve ICB response is to activate the innate immune system via pattern recognition receptors (PRRs) (Datta et al., 2023), leading to Types I and II IFNs in the tumor microenvironment that promote tumor control by directly inducing cell death or enhancing antitumor immunity. Double-stranded RNA, typically associated with viral infection, potently activates Type I IFNs via innate immune receptors, such as melanoma differentiation-associated protein 5 (MDA5), protein kinase R (PKR) and Z-DNA binding protein 1 (ZBP1) (Levanon et al., 2024). Besides, endogenous IR-Alu sequences or cis-natural antisense transcripts (cis-NATs) are sources of self-dsRNA that elicits type I IFNs response by activating PKR (Jiao et al., 2022; Kuriakose et al., 2016; Li et al., 2022b; Thapa et al., 2016; Upton et al., 2012). Z-RNA, a left-handed helix of dsRNA, binds to ZBP1 receptor-interacting protein kinase 3 (RIPK3) to induce caspase 8-dependent apoptosis, mixed lineage kinase domain-like protein (MLKL)-dependent necroptosis, and the nucleotide oligomerization domain-like receptor family pyrin domain containing 3 (NLRP3)-gasdermin D (GSDMD)-dependent pyroptosis (Fig. 2J) (Jiao et al., 2022; Kuriakose et al., 2016; Thapa et al., 2016; Upton et al., 2012). In this connection, ADAR1 isoform plays a gatekeeper role through A-to-I editing of endogenous IR-Alu elements in self-dsRNA and Z-RNA, which prevents self-dsRNA to activate PKR and ZBP1. Indeed, it has been proposed that IFN-inducible expression of ADAR1 acts as a negative-feedback mechanism to counteract the increased responsiveness to self-dsRNA during inflammation (Fig. 2K and 2L) (Chen and Hur, 2022; de Reuver and Maelfait, 2023). Concordantly, the deletion of ADAR1 in tumors cells led to elevated antiviral cytokines and chemokines in response to IFN stimulation. Sustained activation of dsRNA sensor pathway by ADAR1 deletion also reduces cancer cell viability, as shown by the application of anti-cancer epigenetic inhibitors capable of inducing the transcription of repetitive sequences that form dsRNAs (Fig. 5G) (Chen et al., 2021; Chung et al., 2018; Liu et al., 2019). Hence, a number of studies have explored whether ADAR1 blockade potentiates immunologic response in the context of ICB therapy.

Ishizuka et al. (2019) utilized an *in vivo* clustered regularly interspaced short palindromic repeats (CRISPR) screen for the unbiased identification of targets that synergize with ICB therapy and revealed ADAR1 as the top candidate gene selectively depleted in B16 murine melanoma in immunocompetent mice plus anti-PD-1 treatment as compared to mice lacking T cells. Mechanistically, ADAR1 depletion or mutation resulted in impaired A-to-I editing of dsRNA, leading to the activation of MDA5 and PKR receptors, which respectively promote immune infiltration and inhibit tumor growth. Loss of ADAR1 reversed anti-PD-1 resistance in antigen presentation-deficient, but IFN-sensitive, tumors by eliciting inflammation. Another study demonstrated that ADAR1 depletion triggered Z-RNA accumulation and activation of ZBP1–RIPK3-dependent necroptosis (Zhang et al., 2022b). As ADAR1 inhibitor is not available, Zhang et al. (2022b) identified a small molecule, curaxin CBL0137, which directly activates ZBP1 by provoking Z-DNA formation (Fig. 5H). In animal models of melanoma, CBL0137 reverses anti-PD-1 resistance by promoting ZBP1-RIPK3-dependent necroptosis and driving CD8^+^ T cell recruitment (Zhang et al., 2022b). ADAR1 also inhibits ZEB1-mediated PANoptosis (inflammatory cell death pathway) to promote CRC and melanoma in mice via its action on dsRNA (Karki et al., 2021).

Apart from attenuating dsRNA response, ADAR1 unleashes immunosuppressive lncRNA LINC00624 via A-to-I editing. LINC00624 suppresses major histocompatibility complex class (MHC) I antigen presentation and limits CD8^+^T cell infiltration in tumor microenvironment. Moreover, LINC00624 propagates a positive feedforward cycle by promoting stabilization of ADAR1 by inhibiting its ubiquitination-induced degradation via beta-transducin repeat-containing protein (β-TrCP), leading to resistance towards ICB and anti-human epidermal growth factor receptor 2 (HER2) treatment (Fig. 5I) (Zhang et al., 2022a). A-to-I editing-independent immunomodulatory effects of ADAR1 have also been reported. In GC, ADAR1 suppresses IFN signaling in GC through miR-302a-IRF9-STAT1 signaling thereby abrogating antitumor immunity (Fig. 5J) (Jiang et al., 2020). Lenalidomide is a highly effective drug against multiple myeloma by multiple mechanisms including immunomodulation, anti-angiogenesis (Kotla et al., 2009) and CRL4^CRBN^ E3 ligase inhibition (Fink and Ebert, 2015). ADAR1 was shown to induce immunomodulatory drug lenalidomide resistance in multiple myeloma by enhancing Alu-dependent editing and transcriptional activities of GLI1 (arginine-to-glycine substitution) (Lazzari et al., 2017), a Hedgehog (Hh) signaling pathway activator and self-renewal agonist (Fig. 5K). Taken together, ADAR1 is a promising immune therapeutic target for improving ICB therapy in cancer.

## Targeting A-to-I modification

### Pharmacological targeting of aberrant A-to-I modification via ADAR1 inhibition

#### Direct targeting of ADAR1

Multiple efforts have been made to develop small molecule inhibitors of A-to-I enzymes, especially ADAR1. A comprehensive high-throughput screening was performed to identify potential inhibitors of ADAR1 and two compounds, lithospermic acid and Regaloside B, which interact with ADAR1 Zα domain (Hong et al., 2024), the functional domain that binds to Z-RNA and Z-DNA. In another study, alendronate, etidronate, and zoledronate were shown to inhibit the ADAR1 Zα domain through interacting with Lys169, Lys170, Asn173, and Tyr177 of the Zα domain in a similar fashion to the helical backbone of Z-RNA (Choudhry, 2021). Short RNA duplexes incorporating nucleoside analog 8-azanebularine has also been shown to selectively inhibit ADAR1, but not ADAR2 (Fig. 5L) (Mendoza et al., 2023).

#### Targeting ADAR1 splicing and nuclear export

ADAR1 isoform switching into ADAR1p150, a highly active A-to-I isoform, is a potential target to inhibit ADAR1 activity in cancer. Rebecsinib is the first such inhibitor reported (Crews et al., 2023), and it was shown to bind the spliceosome core complex and impair ADAR1p150 activation. By inhibiting ADAR1p150-mediating A-to-I editing, Rebecsinib impaired the self-renewal of LSC *in vitro*, and prolonged survival of humanized mice harboring LSC *in vivo*. Moreover, Rebecsinib spared normal hematopoietic stem and progenitor cells, and it showed favorable toxicokinetic and pharmacodynamic properties.

An alternative strategy is to block the nuclear export of ADAR1p150, which predominantly resides in the cytoplasm to catalyze A-to-I conversion. As the N-terminus of ADAR1p150 contains a NES as mentioned above, treatments targeting nuclear export of ADAR1p150, such as selective nuclear export inhibitors (NEIs), have been reported to be beneficial in cancer treatment (Azizian and Li, 2020; Gravina et al., 2014). NEIs such as leptomycin B, Selinexor (KPT-330), and Eltanexor (KPT-8602) have antitumor efficacy in preclinical models. KPT-330 has recently received US FDA approval for relapsed/refractory multiple myeloma (Chari et al., 2019; Karki et al., 2021; Theodoropoulos et al., 2020). Additionally, the use of IFNs to induce ZBP1, a Z-RNA sensor normally sequestered by ADAR1, could exacerbate induction of cell death when ADAR1p150 is targeted by NEIs. Mechanistically, KPT-330 and KPT-8602 inhibition competes with ADAR1p150 and allowing induction of cell death via ZBP1. Consequently, targeting ADAR1p150/ZBP1 axis by the combination of NEIs and IFN therapy regressed tumors by inducing PANoptosis (Fig. 5M) (Serafimova et al., 2012; Vercruysse et al., 2017).

### Harnessing ADAR1 for site-directed editing by CRISPR/Cas9

CRISPR-Cas systems have been widely employed to edit and modify specific nucleotides on DNA and RNA. Through taking advantage of the A-to-I regulators, a series of strategies have been proposed to manipulate A-to-I modification at specific RNA sites. Site-directed RNA editing by ADAR targets-specific transcripts by generating guanosine mismatches (Montiel-González et al., 2016; Yuan et al., 2023). Unlike DNA editing, RNA A-to-I editing does not impart a permanent modification to the host genome, which confers significant safety benefits and mitigates the side effects of off-target editing that may occur (Li et al., 2022a).

Although several site-directed editing strategies have been reported, they nonetheless share the same basic framework involving re-direction of ADAR deaminase domain to a specific region of the genome. To achieve site-directed RNA editing, a guide RNA (gRNA) is required to direct ADAR enzyme to the specific target site where editing is intended. The most common approach uses genetically engineered ADAR deaminase domain (ADARDD) that binds to a guide RNA. Exogenous ADAR proteins or their catalytic structural domains are fused to CRISPR-Cas13 (Tong et al., 2023; Xu et al., 2021), λ phage N protein (Austin et al., 2002; Baron-Benhamou et al., 2004; Keryer-Bibens et al., 2008; Montiel-Gonzalez et al., 2013), SNAPtag (Stafforst and Schneider, 2012; Vogel et al., 2014) or, and chimeric ADAR proteins (such as SNAP-ADAR) are then directed by sgRNA to the target sites to mediate A-to-I modification. These approaches differ in the mechanism that couple gRNA to ADARDD. For instance, CRISPR13b strategy utilizes catalytically dead Cas13b fused to N-terminal of ADARDD from ADAR1/2, which are co-delivered with gRNA composed of a 50 nt 5ʹ region complementary to the target sequence and containing a C mismatch to the targeted adenosine nucleotide. However, a pitfall in this approach is the need to deliver genetic engineered ADARDD-containing proteins, which might elicit undesirable immune responses in clinical applications. An alternative approach aiming to avoid the pitfalls of exogenous ADARDD by recruiting endogenous ADAR1/2 for targeted editing. Leveraging Endogenous ADARs for Programmable Editing of RNA (LEAPER) and CLUSTER are two systems developed to take advantage of endogenous ADAR, but their gRNAs differ. LEAPER is designed based on the premise that a long gRNA (~70 nt with a single C to A mismatch) could anneal target transcripts to form dsRNA substrates that are recognized by endogenous ADARs, whereas the gRNA for CLUSTER is a cluster guide RNA combining a specificity domain (20 nt with a single C to A mismatch), ADAR recruiting domain (R/G-motif), and a cluster of recruitment sequences (RS) (Katrekar et al., 2022; Qu et al., 2019; Reautschnig et al., 2022; Yi et al., 2022). Further modifications to sgRNA could enable light-triggered site-specific RNA A-to-I editing, leading to light-dependent point mutation of mRNA transcripts inside living cells and 3D tumorspheres in a spatially-defined manner. This represents a new approach for precise manipulation of A-to-I RNA editing in cancer treatment (Fig. 5N) (Zhang et al., 2023).

## Future perspectives and conclusion

Many lines of evidence linking A-to-I editing aberrantly expressed to cancer strongly suggest that developing inhibitors targeting its pathways will be a fruitful pursuit. The A-to-I regulator ADAR1 is significantly overexpressed and promotes tumorigenesis in many cancer types, and high expression of ADAR1 often predicts poor survival in these patients, whereas ADAR2 is more frequently ascribed with a tumor suppressive role. Nevertheless, A-to-I RNA modification appears to serve as a double-edged sword in tumor development, the function of ADAR1 and ADAR2 is often cancer type-specific and context-dependent. More in-depth investigations in physiologically relevant models of cancer, such as ADAR1 or ADAR2 conditional knock-in/knockout mice, will unravel collectively impact of deletion of ADARs *in vivo* on tumorigenesis.

While our understanding of the biology of ADARs-mediated A-to-I editing on mRNA and noncoding RNA has rapidly expanded, the physiological and pathological significances of other A-to-I editing processes, such as the A-to-I editing of tRNAs, are only beginning to be explored. In humans, it has been reported that A-to-I modifications, both at Positions 34 and 37 of tRNA^Ala^, are important for the recognition of autoantibodies generated against the anticodon stem loop of tRNA^Ala^ in patients suffering from myositis, a chronic inflammatory muscle disorder (Becker et al., 1999). However, their potential roles in tumorigenesis remain unexplored. The diversity of tRNA mutations and multiple diseases in which they are involved gives hope that this is only the beginning of the era of tRNA editing in cancer.

From a translational and clinical perspective, it will be of interest to further investigate the use of ADAR blockers for cancer treatment and combating chemoresistance, especially for ADAR1, in light of its predominant pro-tumorigenic effect in multiple studies. In particular, the combination ADAR1 inhibitors with immune checkpoint therapies to unleash antitumor immune response is another area of active investigation. Nevertheless, the development of ADAR-isoform-specific inhibitors is still underexplored. Additional preclinical studies will be needed to establish the efficacy of ADAR blockade for improving cancer therapy. Besides, whether ADAR expression or their downstream A-to-I modifications might be biomarkers for predicting cancer diagnosis or prognostication also deserve further investigations.

In conclusion, the A-to-I modification is involved in a variety of pathological processes, especially tumorigenesis. However, our understanding of A-to-I modification regulators is not yet comprehensive. Only two groups of deaminases complexes, ADARs and ADATs, have been identified to date, and there remain many questions regarding the intricate process of A-to-I modification. Firstly, it is unclear whether A-to-I editing is a dynamic and reversible process or whether there exist corresponding erasers that regulate the balance of A-to-I modification. Secondly, it is unknown whether ADAT2/3 regulates tumorigenesis by affecting the genome-wide codon use preference and protein translation efficiency of tRNA as mentioned above. Additionally, how many new viable codons can be generated by targeted mRNA editing? Do cells use this strategy to modulate protein diversity? There is no doubt that addressing these questions will require a great deal of work. However, the results generated from these experimental efforts will significantly advance our knowledge of the breadth of A-to-I function. Since the posttranscriptional network is intricate and various regulators are often connected, it is worthwhile exploring whether A-to-I and other posttranscription editing influence each other cooperatively to play a greater number of roles, especially in tumors. Further mechanistic studies are imperative to begin to unravel these mysteries.
